# Heavy Metal Enrichment in Ferromanganese Nodules and Soil Ecological Risk Assessment in the Karst Area with High Geological Background

**DOI:** 10.3390/toxics13090746

**Published:** 2025-08-31

**Authors:** Xiangru Zhang, Yifang Su, Haoyi Wang, Shuang Lü, Jinru Su, Guanyu Wei, Haini Huang

**Affiliations:** 1 Guangxi Key Laboratory of Environmental Processes and Remediation in Ecologically Fragile Regions, Guangxi Normal University, Guilin 541004, China; 2 College of Environment and Resources, Guangxi Normal University, Guilin 541004, China; 3 Key Laboratory of Ecology of Rare and Endangered Species and Environmental Protection, Guangxi Normal University, Ministry of Education, Guilin 541004, China

**Keywords:** heavy metals, ferromanganese nodules, karst area, ecological risk

## Abstract

Ferromanganese nodules exhibit strong capacity for heavy metal immobilization and are thus a crucial contributor to the high geological background in karst areas. Heavy metals sequestered within ferromanganese nodules display low bioavailability, which leads to an overestimation of ecological risk in areas with high geological backgrounds. However, limited attention is given to the enrichment process of heavy metals and the overestimated ecological risk of ferromanganese nodules in karst areas. Here, the surface soils and ferromanganese nodules are collected from a region dominated by carbonate and clastic rocks to investigate the enrichment of heavy metals (Cr, Ni, Cu, Zn, As, Cd, Pb, and Hg), the influence of parent rock, and their ecological implications in Northeastern Guangxi. Results show the following findings: (1) Heavy metals are enriched in ferromanganese nodules, with Cr and As correlating with Fe, and Cd and Pb correlating with Mn. (2) The spatial distribution of each element closely matches parent rock lithology, and high heavy-metal concentrations of both soils and ferromanganese nodules occur in carbonate areas. (3) The proportion of contaminated samples generally decreases after excluding the contribution of ferromanganese nodules, leading to a decline in risk level in carbonate areas, while clastic areas exhibit minimal change.

## 1. Introduction

Heavy metals usually refer to metallic elements with a specific gravity greater than 5 g/cm^3^ [1]. Those include necessary elements such as Iron (Fe), Manganese (Mn), Copper (Cu), Zinc (Zn), and Nickel (Ni), which are beneficial to human health at appropriate concentrations, as well as toxic pollutants such as Lead (Pb), Arsenic (As), Mercury (Hg), Chromium (Cr), and Cadmium (Cd), which can accumulate in living organisms and may have negative impacts on the environment and human health [2]. In recent years, irrational human activities such as sewage irrigation, the use of pesticides and fertilizers, leaching of solid waste, and mining have increasingly exacerbated the problem of heavy metal soil pollution both domestically and internationally [3,4,5,6,7,8]. This kind of pollution not only directly leads to the decline of soil quality, but also further causes the excessive content of heavy metals in crops, which ultimately endangers human health through the food chain [9,10,11]. Therefore, regular monitoring and scientific management of heavy metals in soil are crucial for protecting the environment and promoting human health.

Although the increase in heavy metal concentrations in soil is mostly due to irrational human activities, the abnormal enrichment of heavy metals in karst areas is caused by natural factors [12,13,14]. In these areas, karst soils typically exhibit elevated geological backgrounds, caused by the inheritance of parent rock and the pedogenesis process. As the soil volume shrinks sharply during pedogenesis, heavy metals are leached from the parent rock and subsequently bound to ferromanganese oxides, clay minerals, and organic matter, resulting in a higher concentration of soil heavy metals in karst areas than that in non-karst areas [15,16,17,18,19,20,21,22].

Studies in these karst areas with a high geological background show a significant relationship between the enrichment of heavy metals and ferromanganese nodules whose formation is accompanied by weathering and pedogenic processes [17,18,19]. Driven by fluctuating redox conditions and alternating adsorption−precipitation reactions, Fe and Mn ions in the soil solution precipitate and continuously accumulate around nucleation centers, resulting in the formation of ferromanganese nodules and the enrichment of heavy metals [23]. Previous studies have mainly focused on the physicochemical properties and formation processes of ferromanganese nodules, whereas the mechanisms underlying their adsorption of heavy metals as well as the geochemical characteristics derived from different parent rock regions remain insufficiently understood [17,18,19,23,24,25,26,27,28]. In addition, studies on the ecological risks of heavy metals in soil-crop systems have shown that, despite the significantly higher heavy metal content in soils and ferromanganese nodules compared to the regional soil background, the exceedance rate of heavy metals in associated crops has not increased, indicating relatively low bioavailability [20,29,30,31,32,33,34]. Therefore, it is crucial to account for the impact of heavy metals in ferromanganese nodules when assessing the ecological risk of soils in high geological background areas [17].

The southwestern karst region of China, including Guangxi, Guizhou, and Yunnan Provinces, is the largest continuous exposed karst region in the world, covering an area of about 620,000 km^2^, which is approximately 6.5% of China’s total land area [17,19,20]. The high geological background of soil heavy metals in this region has attracted widespread attention [12,13,14,15,16,17,18,19,20,21,22]. In this research, a study area in Guilin City, Guangxi Province, Southwestern China, which consists of two adjacent single parent rocks of carbonate rock and clastic rock, was selected to determine the concentrations of toxic heavy metals (Cr, Ni, Cu, Zn, As, Cd, Pb, and Hg) in surface soil and ferromanganese nodules. The specific objectives are to achieve the following: (1) clarifying the enrichment mechanisms of different heavy metals in ferromanganese nodules; (2) distinguishing the effects of parent rocks on the heavy metals of soil and ferromanganese nodules; and (3) correcting the ecological risk of soils and carrying out ecological risk assessment of heavy metals targeting the soil matrix in high geological background areas. The research is beneficial for understanding the migration and evolution patterns of heavy metals in karst soil, and avoiding the misjudgment of ecological risks due to geological background factors, thereby providing an effective reference for the risk prevention and scientific management of soil heavy metal pollution.

## 2. Description of the Study Area

**Figure 1:**
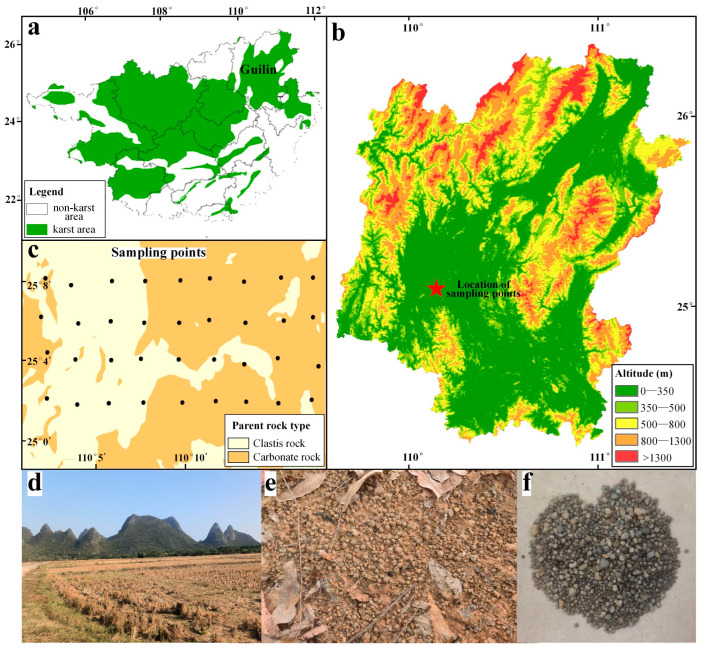
Maps showing the study area: (**a**) the location of Guilin in Guangxi; (**b**) the topographic map of Guilin; (**c**) the locations of sampling points; (**d**) main geomorphic types of the study area; (**e**) ferromanganese nodules in surface soil; (**f**) ferromanganese nodules after selection.

The study area is located in Guilin City, Guangxi Province (109°36′50″–111°29′30″ E, 24°15′23″–26°23′30″ N) (Figure 1a,b). The tectonic framework of the Guilin area is essentially established during the Late Paleozoic Era [35]. Although tectonic events occur in the western part of Guangxi since the Mesozoic-Cenozoic Era [36], their impact on the northeastern area of Guilin is limited. From the perspective of the modern tectonic framework, there is little fault activity in the northeastern region of Guangxi, including the Guilin area [37,38]. This region is characterized by typical karst landforms such as karst peak forests and cone karst depressions (Figure 1d). The climate is subtropical monsoon, with a mild temperature (the average annual temperature is close to 19.1 °C), abundant rainfall (the average annual rainfall is 1887.6 mm), a long frost-free period (285 days), sufficient sunshine (the average annual sunshine duration is 1447.1 h), distinct seasons, and rain and heat occurring in the same season [39].

The samples were collected from a karst basin in the southwestern part of Guilin City (110°1′48″–110°18′9″ E, 25°1′47″–25°8′6″ N) (Figure 1c), within which no distinct fault zones were observed [37,38]. This region includes two adjacent single parent rocks of carbonate and clastic rocks. The strata from bottom to top are the Upper Paleozoic, the Middle and Upper Devonian, the Lower Carboniferous, the Upper Triassic of the Mesozoic, the Upper Cretaceous, and the Tertiary and Quaternary of the Cenozoic. The Devonian and Carboniferous carbonate rocks are widely distributed, featuring typical platform shallow-water and platform slope sedimentary types [40]. The land in the study area is mainly agricultural, with crops such as citrus and rice commonly seen, and no mining activities have been observed.

## 3. Sample Collection and Analytical Method

### 3.1. Sample Collection and Ferromanganese Nodules Selection

To ensure the uniform distribution of sampling points, a regular grid system for sampling was used. Within the designated sampling area, samples were collected at a density of 2 km^2^ per point, resulting in a total of 32 surface soil samples (0–20 cm). All samples were collected using a shovel and then sealed in airtight plastic bags, which were subsequently neatly arranged into wooden boxes to avoid physical impacts during transportation. Based on the different types of bedrock underlying the weathering crust, the collected surface soil samples were divided into two categories: clastic rock and carbonate rock areas. The locations of the sampling points are shown in Figure 1c. Sampling was carried out from November to December 2024 (about 20 °C), and the samples were delivered to the laboratory with a constant temperature (22 °C) on the day of collection.

In the laboratory, after removing plant residues, pebbles, and other components, the field-collected samples were naturally air-dried in a cool, ventilated, clean, and dust-free environment. Subsequently, each soil sample was divided into two equal-weight portions. One portion was designated as the soil sample, and the other portion was selected for ferromanganese nodules. For the latter part of the soil samples, large nodules visible to the naked eye (>2 mm) were first manually picked out. The remaining samples were then placed into a 50-mesh nylon sieve for wet sieving. Subsequently, smaller nodules particles (0.3 mm–2 mm) were carefully picked out from the sieved materials using plastic tweezers and a magnifying glass. Lastly, all the nodules were washed with deionized water and then air-dried. Particles finer than 0.3 mm, which were difficult to manually pick out, were not considered in this study. The selected nodules were characterized by dark color, hard texture, and a rounded structure (Figure 1f). The selection process of ferromanganese nodules refers to the methods by Sun et al. [26] and Ettler et al. [41], with modifications and enhancements to improve the method. The mass and proportion of ferromanganese nodules are shown in Supplementary Table S1. Finally, all samples were ground with a mortar to prepare for analysis and testing.

### 3.2. Chemical Analysis

Soil samples and the selected ferromanganese nodule samples were accurately weighed at 40 mg. After adding 2 mL of mixed acid (concentrated HNO_3_ and concentrated HF (1:3)), each sample was placed in a closed autoclave for digestion. Additionally, a blank sample was added to the experiment during the digestion process to ensure that no impurities were introduced. The blank sample was treated in the same way as the digestion process, with the exception that no soil sample was weighed out. HNO_3_ and HF were also added to the blank sample. The digested samples were then diluted twice with 2% purified HNO_3_ to an appropriate multiple. The acidified aqueous phase after dilution was stored in glassware and tested immediately after digestion to prevent the adsorption of mercury in a container. The detailed operating procedures refer to Yang et al. [42]. Inductively coupled plasma mass spectrometry (ICP-MS) was used to determine the elements such as Cr, Ni, Cu, Zn, As, Cd, Pb, Hg, Fe, and Mn. The sample analysis and testing were completed at the School of Earth Sciences, Guilin University of Technology. A total of 32 soil samples and 32 ferromanganese nodule samples were analyzed in this study. A blank sample (ultrapure water) was immediately measured after every 12 samples were measured, in order to keep the experimental data within a reasonable error range. Additionally, two rock reference samples (GSR-1 and GSR-3) were tested in this study. The test results were within the error range, ensuring the accuracy of the entire experimental process, including digestion and testing.

### 3.3. Data Processing

#### 3.3.1. Enrichment Coefficient (*EF*)

The enrichment coefficient (*EF*) is generally utilized to signify the degree of enrichment of an element relative to its natural source [43]. To characterize the enrichment degree of various heavy metal elements in ferromanganese nodules, the enrichment factor (*EF*) is obtained by comparing the concentration of heavy metal elements in ferromanganese nodules (*C_nodules_*) with that in the soil matrix (*C*_soil_) [44]:

*EF* = *C_nodules_*/C_soil_ (1)

In this study, *C*_soil_ is calculated based on the mass conservation formula:

*M*_total_ × *C*_total_ = *M*_soil_ × *C*_soil_ + *M*_nodules_ × *C*_nodules_ (2)

where *M*_total_ refers to the mass of the soil sample; *M*_soil_ refers to the mass of soil matrix after the removal of ferromanganese nodules from the soil sample; *M*_nodules_ refers to the mass of ferromanganese nodules; *C*_total_ refers to the concentration of heavy metal in the soil sample; *C*_nodules_ represents the concentration of heavy metals in ferromanganese nodules.

When the *EF* value is >1, it indicates that the element is enriched in the ferromanganese nodules. Conversely, it indicates that the element is depleted in the ferromanganese nodules.

#### 3.3.2. Pollution Risk Assessment

The ecological risk of active heavy metals is assessed as follows.

1. Single Factor Index (*SFI*)

The pollution index is calculated by removing the scale from the measured value of the heavy metal element compared to the evaluation criteria, using the formula [45,46]:

(3) $$\;P_{i}=\frac{C_{i}}{S_{i}}$$

where $P_{i}$ is the pollution index of the heavy metal element *i*, $C_{i}$ is the measured value of the heavy metal element, and $S_{i}$ is the soil environmental quality standard value of heavy metal element *i* (national secondary standard value).

2. Nemerow Comprehensive Pollution Index (*NCPI*)

The Nemerow Comprehensive Pollution Index method takes into account both the Single Factor Index and the Nemerow Index, highlighting the role of the more polluting heavy metal pollutants. It can be calculated as follows [46]:

(4) $$N=\sqrt{\frac{P_{ave}^{2}+P_{max}^{2}}{2}}$$

where *N* is the comprehensive pollution index of a single sampling point, $P_{ave}$ is the average value of the single factor index, and $P_{max}$ is the maximum value of the single factor index.

Due to the different impacts of different heavy metal elements on the soil environment and ecosystem, a weighted calculation method is used to find the average value, with the following formula:

(5) $$\;{\;\;\;P}_{ave}=\frac{\sum_{i=1}^{n}W_{i}P_{i}}{\sum_{i=1}^{n}W_{i}}$$

where $W_{i}$ is the weight of the heavy metal element *i*, determined based on the following improved *AHP* method [47]:

The three-scale method is used to construct the comparison matrix $B={\left(b_{ij}\right)}_{n\times n}$. Based on the relative magnitudes of the limit value of various heavy metal elements in grain crops specified in the National Standard of the People’s Republic of China, $b_{ij}$ is defined as:

(6) $$b_{ij}\left\{\begin{array}{c} 2\;\mathrm{The}\;\mathrm{limit}\;\mathrm{value}\;\mathrm{of}\;\mathrm{element}\;i\;\mathrm{is}\;\mathrm{greater}\;\mathrm{than}\;\mathrm{the}\;\mathrm{limit}\;\mathrm{value}\;\mathrm{of}\;\mathrm{element}\;j.\\ 1\;\mathrm{The}\;\mathrm{limit}\;\mathrm{value}\;\mathrm{of}\;\mathrm{element}\;i\;\mathrm{is}\;\mathrm{equal}\;\mathrm{to}\;\mathrm{the}\;\mathrm{limit}\;\mathrm{value}\;\mathrm{of}\;\mathrm{element}\;j.\\ 0\;\mathrm{The}\;\mathrm{limit}\;\mathrm{value}\;\mathrm{of}\;\mathrm{element}\;i\;\mathrm{is}\;\mathrm{less}\;\mathrm{than}\;\mathrm{the}\;\mathrm{limit}\;\mathrm{value}\;\mathrm{of}\;\mathrm{element}\;j. \end{array}\right.$$

Calculate $r_{i}$ = Σ$b_{ij}$(i = 1, 2......n), i.e., sum by row, and then use Formula (7) to obtain the decision matrix *C* $={\left(c_{ij}\right)}_{n\times n}$. Solve for the maximum eigenvalue and eigenvector of the heavy metal elements using the decision matrix *C*. After passing the consistency test, standardize the eigenvector to obtain the weights of the heavy metal elements:

(7) $${\;\;C}_{ij}=\left\{\begin{array}{c} {}[(r_{i}-\;r_{j})/(r_{max}-\;r_{min})]\times (b_{m}-\;1)+1\;\;\;\;\;\;\;r_{i}\geq r_{i}\\ {}[(r_{j}-\;r_{i})/(r_{max}-\;r_{min})]\times (b_{m}-\;1)+1\}-1\;\;\;\;\;r_{i}<r_{j} \end{array}\right.$$

where $r_{max}$ = Max{$r_{i}$}, $r_{min}$ = Min{$r_{i}$}, and $b_{m}$ = $r_{max}{/r}_{min}$.

3. Potential Ecological Risk Index (*PERI*)

The Potential Ecological Risk Index (*PERI*) method evaluates heavy metals in soil or sediment from a sedimentological perspective, based on the nature of the heavy metals and their behavioral characteristics, such as transport, transformation, and deposition in the environment. The calculation formula is as follows [46,47,48]:

(8) $${\;\;\;E}_{r}^{i}=T_{i}\times \frac{C_{i}}{C_{o}}$$

(9) $$RI=\sum_{i=1}^{n}E_{r}^{i}$$

where $E_{r}^{i}$ is the potential ecological risk index of heavy metal element *i*; $C_{i}\;$ is the measured value of heavy metal element *i*; $C_{o\;}$ is the reference value, and the geochemical reference value of soil in the region is used; $T_{i}$ is the toxic-response factor of metal element *i*, and the values are Hg = 40, Cd = 30, As = 10, Pb = Cu = Ni = 5, Cr = 2, and Zn = 1 [46]. The *RI* is the combined index of potential ecological risk of multiple heavy metals. Based on the values of $E_{r}^{i}$ and RI, the potential risk levels of individual heavy metal elements and multiple heavy metal elements in soil are determined separately.

The above pollution indices grading standards for each method are shown in Table 1.

## 4. Results

The results of the heavy metal concentrations in soils are shown in Table 2. Among them, the average concentration of heavy metals is the highest for Zn (156.15 mg/kg) and the lowest for Hg (0.12 mg/kg). The average concentrations of Cr, Ni, Pb, Cu, Zn, As, Cd, and Hg in the soil are 1.85, 1.94, 1.45, 2.07, 1.47, 2.15, 1.66, and 0.80 times the soil background of Guangxi, respectively. At the same time, these elements also far exceed the national soil background values, showing abnormal accumulation of Cr, Ni, Zn, As, Cd, and Pb. The variation coefficient of each heavy metal element is in the order of Hg > As > Ni > Cr > Cd > Cu > Pb > Zn, with Hg (1.14) showing high variation, indicating that Hg is unevenly distributed in the study area, while the other heavy metal elements show moderate variation. In addition, the concentrations of heavy metals in the carbonate region are higher than those in the clastic region, with the most significant differences in Cr and Hg.

At the same time, the average concentrations of Cr, Ni, Cu, Zn, As, Cd, Pb, and Hg in the ferromanganese nodules are 1093.88, 77.64, 45.05, 272.80, 169.99, 1.06, 79.16, and 0.2 mg/kg, respectively (Table 3). All of the *EF* values are greater than 1, indicating that heavy metals in the soil tend to be enriched in ferromanganese nodules. The heavy metals in the nodules of the carbonate area are also significantly higher than those in the clastic rock area.

## 5. Discussion

### 5.1. The Enrichment of Heavy Metals in Ferromanganese Nodules

Ferromanganese nodules in soils form during weathering and pedogenesis through the repeated redox reactions and dissolution−precipitation of Fe and Mn. These elements co-aggregate with other soil constituents into concentrically banded structures [23]. Owing to their high specific surface area, redox activity, distinctive microstructure, multi-mineral synergy, and microbial mediation, the nodules exhibit exceptional heavy-metal immobilization capacity [23]. In this study, all heavy metals, except Cu, which are similar to the soil matrix, are markedly enriched in ferromanganese nodules. Concentrations of Cd, Zn, Pb, Ni, and Hg are roughly twice those in the soil matrix, whereas Cr and As reach 8.55 and 6.63 times the matrix levels, respectively. This underscores the critical role of ferromanganese nodules in concentrating heavy metals in karst regions. The soil in the Guangxi region contains abundant ferromanganese nodules, which is an important reason for the high heavy metal content in the karst areas of Guangxi [50].

Previous studies have shown that, owing to the differences in electron configuration, ionic radius, electronegativity, and hydrolysis capacity, distinct heavy-metal ions bind to ferromanganese nodules in a markedly element-specific manner [23,24,27,51,52,53,54]. Specifically, Ni^2+^ tends to selectively occupy lattice-defect sites in ferromanganese oxides, whereas Zn^2+^ is partitioned via adsorbing onto ferromanganese oxide surfaces or entering the lattices of layered silicates [51]. Enrichment of Cu and Pb in the nodules is mainly driven by strong adsorption onto manganese oxides and isomorphic substitution of Mn^2+^ [52]. The immobilization of Cd^2+^ relies on surface complexation, lattice substitution, and coprecipitation with manganese oxides [24]. Different from the above heavy-metal ions, Hg^2+^ is fixed by sulfide complexation and finally converted into HgS precipitates within the nodules [53]. Redox-sensitive elements, represented by Cr and As, perform relatively intricate behaviors. Iron oxides dominate the adsorption and immobilization of highly toxic Cr^6+^ which can be reduced to the less toxic Cr^3+^ by Fe^2+^ [54]; Arsenic is almost exclusively immobilized through the specific adsorption by iron oxides, although manganese oxides can also sometimes contribute to its immobilization by oxidizing As^3+^ [27]. Thus, the affinity of various heavy metals for iron and manganese oxides differs substantially, which is consistent with the Synchrotron X-ray analyses that each element’s distribution coincides with its associated host mineral in two distinct trace-element enrichment zones inside the nodules: Pb, Co, Cu, and Cd are preferentially bound to Mn, whereas Cr and As are chiefly associated with Fe [55,56].

**Figure 2:**
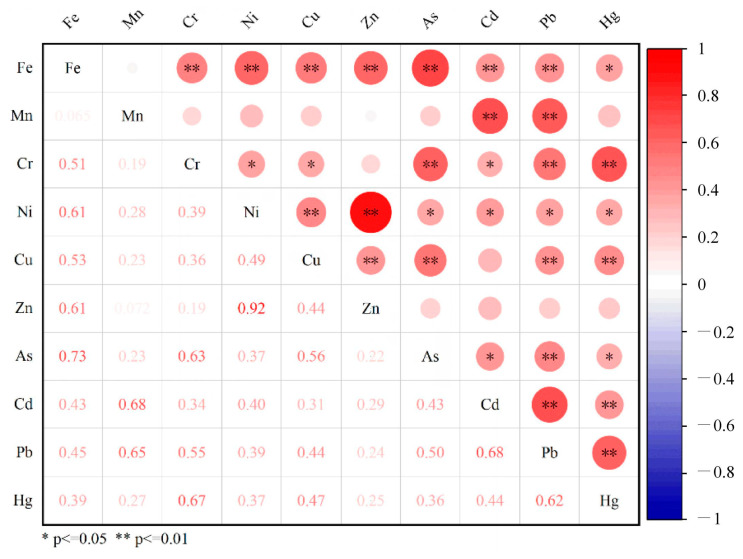
The correlation between heavy metal elements in ferromanganese nodules.

In this study, the correlation analysis of heavy metal elements in ferromanganese nodules (Figure 2) shows certain correlations between Mn and Cd as well as between Mn and Pb, with correlation coefficients of 0.68 and 0.65, respectively. Meanwhile, Fe exhibits strong correlations with As (correlation coefficients of 0.73) and relatively moderate correlations with Ni, Zn, Cu, and Cr (correlation coefficients of 0.61, 0.61, 0.53, 0.51, respectively). This further demonstrates that the enrichment of Cd and Pb in ferromanganese nodules is controlled by the Mn element, while the enrichment of As and Cr is related to the Fe element. Particularly, Ni and Zn show an extremely strong correlation (0.92), which indicates that the chemical behavior and precipitation processes of the two elements are closely related during the formation of ferromanganese nodules, suggesting similar sources and enrichment mechanisms.

The *EF* values of heavy metals in the ferromanganese nodules of the study area are arranged in the following order: Cr (8.55) > As (6.63) > Pb (2.09) > Cd (1.92) > Zn (1.82) > Hg (1.73) > Ni (1.54) > Cu (1.13), with the most significant enrichment of Cr and As (Table 3). This enrichment order is different from that of other typical karst areas, such as Chongzuo in Southwestern Guangxi (Cd > Pb > Cr > Cu > Zn > Ni) [17] and Laibin in Central Guangxi (Cd > Pb > As > Cu > Cr > Ni > Zn) [57], where the enrichment of Cd and Pb is dominant in ferromanganese nodules. This difference is possibly related to the types and contents of iron and manganese oxides in the regional soils. In this study area, the contents of Fe and Mn in nodules are 195.88 g/kg and 2.92 g/kg, respectively, which are 13.7 and 5.3 times those in the matrix soil, respectively. Owing to the relatively higher accumulation of Fe than Mn in the nodules, the Cr and As show higher enrichment levels than Cd and Pb.

### 5.2. The Influence of the Parent Rocks on the Heavy Metal Composition in Ferromanganese Nodules

**Figure 3:**
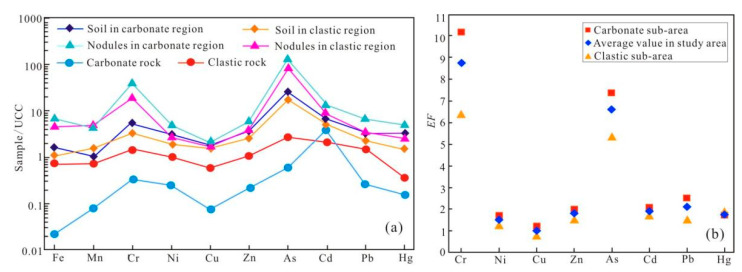
Enrichment characteristics of each element: (**a**) elemental composition normalized to UCC (data of clatic rock and carbonate rock are from Yang et al. [15]); (**b**) ratios of heavy metal concentrations in ferromanganese nodules to those in the soil matrix.

To compare the effects of parent rocks on heavy metals in soils and ferromanganese nodules, the heavy metal elements in parent rocks, soils, and ferromanganese nodules from carbonate and clastic regions are normalized to the upper continental crust (UCC) [58,59]. Results of heavy metals in rocks reveal that the normalized values of all elements in the carbonate rock are lower than those in clastic rock (Figure 3a). All the heavy metals, except for Cd, are relatively depleted in the carbonate rock. The clastic rock shows depletion of Fe, Mn, Cu, and Hg and enrichment of Cr, Ni, Zn, As, Cd, and Pb [15]. The different patterns of heavy metals between carbonate and clastic rocks indicate that all elements are redistributed during the formation of the bedrock. Correspondingly, all heavy metal elements of soils and ferromanganese nodules in both carbonate and clastic regions exhibit higher values, showing a significant enrichment during the subsequent weathering and pedogenesis processes. However, the heavy metal elements in the soils and ferromanganese nodules in different regions present different distribution characteristics (Figure 3a).

For the surface soil, the ratios of all elements to UCC in both carbonate and clastic regions are greater than 1 (Figure 3a). Except for Mn, all the elements show enrichment in the soil of the carbonate rock area, which is consistent with the conclusions drawn by previous researchers [12,13,14,15]. To explain the enrichment mechanism of heavy metals in carbonate region, a two-stage model has been proposed [15]. The first stage is characterized by leaching and accumulation. As the carbonate rocks dissolve and their total volume decreases, the concentration of the leached heavy metals which tend to stay in insoluble residues significantly increases. The second stage is described by “pedogenesis stage” when the insoluble residues are further weathered to soil. A low leaching degree of each heavy metal is observed in this stage, showing a bedrock inheritance of some heavy metals such as Cd, Cr, and Ni. The two-stage model is supported by this study which demonstrates that soil concentrations in the carbonate region are significantly higher than those in the clastic region in Northeastern Guangxi (Figure 3a).

**Figure 4:**
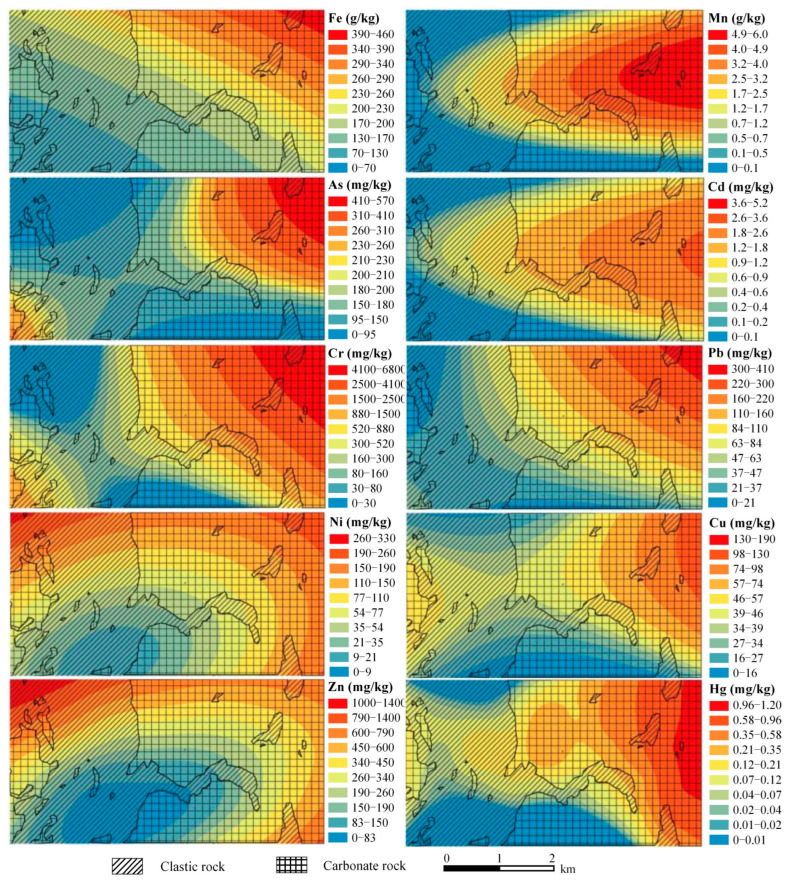
Enrichment characteristics of each element in ferromanganese nodules.

The Fe and Mn in the parent rock act as the basic composition for the formation of nodules. The proportion of ferromanganese nodules is more enriched in the carbonate area (3.88%) compared to in the clastic rock area (1.97%), although the concentrations of Fe and Mn are higher in the clastic bedrock of the study area [15]. This further indicates the enrichment of elements during the weathering and pedogenic processes of carbonates [15,17]. For the heavy metals, ferromanganese nodules exhibit much higher concentrations than parent rock and the soil matrix (Figure 3a,b). Containing two adjacent single-lithology zones (Figure 1c), the study area provides a chance to distinguish the influence of parent rocks on heavy metal enrichment in ferromanganese nodules in the spatial dimension. Based on the spatial interpolation analysis of the 32 sampling points, spatial distribution of heavy metals in the ferromanganese nodules is displayed in Figure 4, which is largely consistent with the spatial distribution of the carbonate and clastic rock sub-regions. In general, the enrichment of heavy metal elements in the nodules of the carbonate region is more pronounced than in the clastic region, while each element displays a spatial specificity to a certain degree. The distribution of Cd in ferromanganese nodules across the entire study area is significantly controlled by the Mn element, showing a decreasing trend from the central-eastern part to the southwest and northwest. Cr and As, on the other hand, show a distribution pattern similar to that of Fe, decreasing from the northeast to the southwest. The distribution patterns of Zn and Ni are highly consistent, both showing a decreasing trend from north to south and from east to west.

### 5.3. Ecological Risk Assessment of Soil Heavy Metals Based on the Correction of Ferromanganese Nodules

Although soil heavy metals in karst regions typically have high geological background, studies have shown that elements such as As, Cr, Zn, Ni, and Cu are overwhelmingly bound in the stable residual fraction (>85%) and therefore exhibit poor mobility, low bioavailability, and limited ecological risk [32]. This is primarily attributed to the presence of ferromanganese nodules, which immobilize heavy metal ions within the residue fraction via adsorption or coprecipitation mechanisms [30]. This general pattern is evident for specific elements like As and Cd. Despite high concentrations in total soils, As^3+^ extracted by HNO_3_ and Cd^2+^ extracted by CaCl_2_ remain low, indicating a restricted transfer to biota [34]. Records that rice and other crops rarely exceed safety limits further corroborate this observation [30,33,34].

Therefore, existing studies primarily relying on bulk soil samples for risk assessment often overlook the critical role of ferromanganese nodules, as geochemically inert components, in controlling heavy metal behavior. This approach may lead to an inaccurate representation of the actual ecological risks posed by soil heavy metals. To more accurately assess the actual pollution status and ecological risks in the study area, the Single Factor Index (*SFI*) method, the Nemerow Comprehensive Pollution Index (*NCPI*) method based on *AHP* (Analytic Hierarchy Process) weights, and the Potential Ecological Risk Index method (*PERI*) were utilized to compare the pollution status of heavy metals in the soils, as well as in the soil matrix after preliminary correction by ferromanganese nodules.

**Figure 5:**
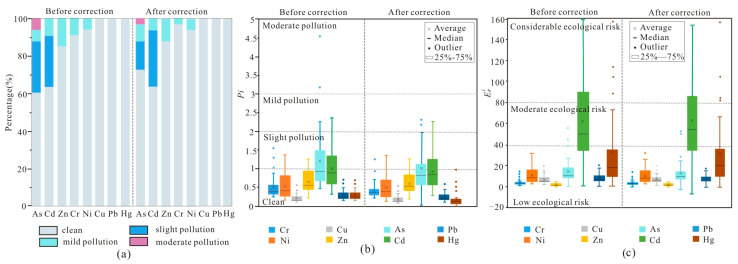
Pollution conditions calculated by different methods: (**a**) pollution proportion of each element by *SFI*; (**b**) *Pi* values of each element; (**c**) $E_{r}^{i}$ values of each element.

The results of the *SFI* calculation show that As, Cd, Zn, Cr, and Ni exhibit pollution at different sampling points in the soils (Figure 5a,b). Among them, As has the highest pollution proportion, followed by Cd, Zn, Cr, and Ni (Figure 5a). The *Pi* values indicate that the heavy metals in the study area are all in a clean state apart from As, showing slight pollution (Figure 5b). After the heavy metals in ferromanganese nodules are excluded, the data distribution range becomes more concentrated, indicating a decrease in the proportion of polluted points (Figure 5b). Although the *Pi* value of As remains slightly above the control line after correction, the proportion of As-polluted sampling points decreases from 39.4% to 17.3%, also demonstrating a reduction in the degree of pollution (Figure 5a,b).

**Figure 6:**
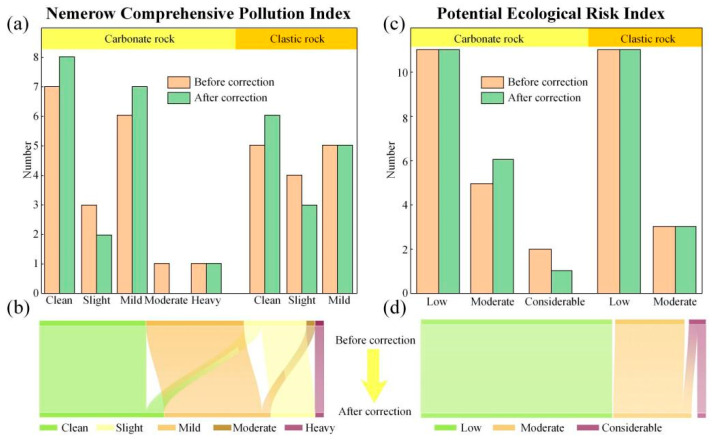
Statistical analysis of sample points and variations in pollution levels before and after correction. (**a**): Sample points at different pollution levels calculated by *NCPI*; (**b**): Variations in sample points before and after correction obtained by *NCPI*; (**c**): Sample points at different pollution levels calculated by *RERI*; (**d**): Variations in sample points before and after correction obtained by *RERI*.

Compared with the evaluation results of the *SFI*, the assessment by the *NCPI* shows that only 36.4% of the soil in the study area is clean, while as high as 57.6% reaches the pollution level (Figure 6a,b). After correction based on ferromanganese nodules, the heavy metal pollution status of the soil samples in the entire area has improved, with 6.06% of the sampling points shifting from the pollution level to clean, increasing the proportion of clean sampling points to 42.42%. Additionally, 3.03% of the sampling points have shifted from mild pollution to slight pollution, indicating a reduction in pollution levels (Figure 6a,b).

Moreover, the average value of the *PERI* for each soil heavy metals $\left(E_{r}^{i}\right)$ is highest for Cd, which is related to its high toxicity coefficient. The data distribution of Cd is also more dispersed, indicating a moderate risk. Other elements do not show pollution characteristics (Figure 5c). The comprehensive *PERI* of multiple heavy metals (*RI*) also indicates that the soil in the study area is contaminated to a certain extent, with more than 30.3% sample points having moderate ecological risk or above (Figure 6c,d). After correction based on ferromanganese nodules, the average $E_{r}^{i}$ values of all heavy metals decrease (Figure 5c). The distribution of Cd still falls within the moderate ecological risk range but becomes more concentrated (Figure 5c). At the same time, the correlation results in 3.03% of sample points shift to moderate risk category from considerable ecological risk category (Figure 6c,d).

Obviously, the overall contamination levels in the soils from the study area are totally overestimated, with the fact that the soil pollution status exhibits a decline after deducting heavy metals within ferromanganese nodules (Figure 5 and Figure 6). However, this overestimation is not statistically significant in this study, possibly resulting from the limited quantity of visible ferromanganese nodules selected for analysis. Moreover, considering that the three assessment methods consistently show a generally low contamination grade across the study area, it is difficult to shift to a lower pollution grade after the correction of ferromanganese nodules.

Furthermore, the heavy metals in the study area exhibit certain spatial differences. Compared with the clastic rock region, the *SFI* and *NCPI* reveal that mild to moderate pollution only occurs in the carbonate rock region, where the proportion of soils with slight pollution is significantly higher, and the *PERI* also indicates that the proportions of sample points with moderate and considerable ecological risk are higher in the carbonate rock region. What is more, the degree of soil contamination significantly decreases after correction in the carbonate region, while the clastic region shows minimal changes. In addition, for specific elements, As and Cd are the principal polluting elements in the study area. Given the enrichment factors of As (6.59) and Cd (1.93) in ferromanganese nodules, correcting for their contribution leads to substantial alleviation in pollution levels.

The soils in the carbonate rock region of Guangxi, China, are generally characterized by high geological background levels. Given the stability of ferromanganese nodules [50], it is necessary to exclude their influence when conducting land-use assessments in karst areas, so that a greater proportion of land can be utilized scientifically and effectively. At the same time, speciation and bioavailability of heavy metals are also needed to systematically resolve the occurrence and release potential of heavy metals in the nodules, and thus accurately understand the pollution-contributing fractions in assessing ecological risk.

## 6. Conclusions

This study reports a relative enrichment of heavy metals in ferromanganese nodules and investigates the potential impact of this enrichment on ecological risk assessment in the high geological background area of Guilin, Guangxi. The results show that ferromanganese nodules exhibit a significant enrichment trend for Cr, As, Pb, Cd, Zn, Hg, and Ni. Weathering and pedogenic processes amplify the enrichment of heavy metals in carbonate regions compared to in clastic rock regions. After deducting the heavy metals in the nodules, the ecological risk level, primarily driven by As and Cd, decreases across the study area. In the carbonate rock region, the risk shifts from mild-to-moderate to slight-to-clean conditions, while clastic rock region exhibits minimal changes. This suggests that the ecological risk of heavy metals based on whole soil samples might be overestimated in carbonate rock regions. Thus, ferromanganese nodules deserve further attention in the ecological risk assessment of heavy metals in karst regions. This study provides useful perspectives that may contribute to ecological risk assessment and rational land-use planning in karst regions. Additionally, further investigation is also needed to account for differences in element speciation and bioavailability.

## Figures and Tables

**Table 1:** Classification criteria for polluted indices of ecological risk assessment [45,46].

Assessment Methodology	Evaluation Criteria					
Single Factor Index	Grade	Clean	Slight pollution	Mild pollution	Moderate pollution	Heavy pollution
	*Pi*	≤1	(1, 2]	(2, 3]	(3, 5]	>5
Nemerow Comprehensive Pollution Index	Grade	Clean	Slight pollution	Mild pollution	Moderate pollution	Heavy pollution
	*N*	≤0.7	(0.7, 1]	(1, 2]	(2, 3]	>3
Potential Ecological Risk Index	Grade	Low	Moderate	Considerable	High	Very high
	$E_{r}^{i}$	<40	[40, 80)	[80, 160]	[160, 320]	≥320
	RI	<150	[150, 300]	[300, 600]	[600, 1200]	≥1200

**Table 2:** Heavy metal concentration in the soils.

	Cr	Ni	Cu	Zn	As	Cd	Pb	Hg
Mean	151.52	51.68	40.31	156.15	30.10	0.58	39.74	0.12
SD	90.14	31.96	19.52	71.68	21.28	0.34	18.83	0.14
Min	70.97	14.31	19.06	50.27	11.56	0.18	19.93	0.00
Max	465.40	136.73	109.30	312.49	113.73	1.42	96.57	0.60
CV	0.59	0.62	0.48	0.46	0.71	0.59	0.47	1.14
Mean value in the carbonate region	175.89	58.20	39.74	170.39	32.67	0.65	43.47	0.15
Mean value in the clastic region	118.45	42.84	41.07	136.82	26.63	0.49	34.69	0.08
Background value of soil in Guangxi [29]	82.10	26.60	27.80	75.60	20.50	0.27	24.00	0.15
Background value of soil in China [49]	70.00	30.00	26.00	73.00	11.00	0.17	28.00	0.06

The coefficient of variation (CV) is unitless, while the units for the other indicators are mg/kg.

**Table 3:** The average concentrations of heavy metals in ferromanganese nodules and the soil matrix.

		Cr	Ni	Cu	Zn	As	Cd	Pb	Hg
Carbonate region	Ferromanganese nodules	1377.15	96.45	47.41	323.35	196.92	1.23	101.50	0.24
	Soil matrix	139.05	56.68	39.24	163.21	27.15	0.61	40.66	0.14
	EF	9.90	1.70	1.21	1.98	7.25	2.00	2.50	1.68
Clastic region	Ferromanganese nodules	709.44	52.12	41.85	204.19	133.43	0.83	48.84	0.14
	Soil matrix	112.81	42.11	40.89	132.65	23.56	0.47	33.95	0.08
	EF	6.29	1.24	1.02	1.54	5.66	1.76	1.44	1.84
Mean	Ferromanganese nodules	1093.88	77.64	45.05	272.80	169.99	1.06	79.16	0.20
	Soil matrix	127.92	50.50	39.94	150.25	25.63	0.55	37.81	0.11
	EF	8.55	1.54	1.13	1.82	6.63	1.92	2.09	1.73

The units for all indicators are mg/kg.

## Data Availability

The data of this study are available on request from the corresponding author.

## Supplementary Materials

The following supporting information can be downloaded at: https://www.mdpi.com/article/10.3390/toxics13090746/s1. Table S1: The mass and proportion of ferromanganese nodules in this study.
