# Supplementary material for: Heavy Metal Enrichment in Ferromanganese Nodules and Soil Ecological Risk Assessment in the Karst Area with High Geological Background
Source: Toxics. 2025 Aug 31;13(9):746. doi: 10.3390/toxics13090746 (PMC12473787; doi:10.3390/toxics13090746)
Supplement: Supplementary file 1 [file toxics-13-00746-s001.zip › toxics-3807765-supplementary.pdf]

toxics

MDPI

Supplementary Table S1. The mass and proportion of ferromanganese nodules in this study.

Sample number	Nodules （g）	Bulk soil (g)	Proportion of nodules(%)	Sample number	Nodules （g）	Bulk soil (g)	Proportion of nod-ules(%)
01	2.9	152.2	1.91	17	0	153.3	0
02	5.5	125.2	4.39	18	0.2	90.3	0.22
03	0	241.8	0	19	0.2	248.6	0.08
04	5.0	182.5	2.74	20	0	191.8	0
05	28.8	245.3	11.74	21	14.6	324.9	4.49
06	4.7	153.7	3.06	22	17.2	287.8	5.98
07	1.5	180.0	0.83	23	26.9	207.5	12.96
08	6.9	207.5	3.33	24	0.8	300.0	0.27
09	2.3	179.4	1.28	25	2.4	306.0	0.78
10	6.1	167.6	3.64	26	0.8	132.8	0.60
11	3.6	258.5	1.39	27	0.6	208.5	0.29
12	5.8	211.6	2.74	28	0	128.1	0
13	13.3	164.1	8.10	29	8.8	286.7	3.07
14	5.0	212.2	2.36	30	0	162.0	0
15	0	122.5	0	31	44.6	202.4	22.04
16	0	89.9	0	32	2.0	165.7	1.21
